# Supplementary material for: Buckwheat and Cardiometabolic Health: A Systematic Review and Meta-Analysis
Source: J Pers Med. 2022 Nov 22;12(12):1940. doi: 10.3390/jpm12121940 (PMC9784502; doi:10.3390/jpm12121940)
Supplement: Supplementary file 1 [file jpm-12-01940-s001.zip › jpm-2026597-supplementary_ELL.pdf]

Supplementary Material

Buckwheat and Cardiometabolic Health: A Systematic Review and Meta-Analysis

Erand Llanaj [1,2,3], Noushin Sadat Ahanchi [4], Helga Dizdari [4], Petek Eylul Taneri [4,5,6], Christa D. Niehot [7], Faina Wehrli [4,8], Farnaz Khatami [4,8], Hamidreza Raeisi-Dehkordi [4,9], Lum Kastrati [4,9], Arjola Bao [4,10], Marija Glisic [4,11] and Taulant Muka [4,2*]

[1] Department of Molecular Epidemiology, German Institute of Human Nutrition Potsdam-Rehbrücke, 14558 Nuthetal, Germany
[2] *Epistudia*, 3012 Bern, Switzerland
[3] ELKH-DE Public Health Research Group of the Hungarian Academy of Sciences, Department of Public Health and Epidemiology, Faculty of Medicine, University of Debrecen, Kassai út 26, 4028 Debrecen, Hungary.
[4] Institute of Social and Preventive Medicine (ISPM), University of Bern, 3012 Bern, Switzerland
[5] School of Nursing and Midwifery, National University of Ireland Galway, Galway, Ireland
[6] HRB-Trials Methodology Research Network, H91 TK33 Galway, Ireland
[7] Literature Searches Support, 3311 Dordrecht, The Netherlands
[8] Department of Community Medicine, School of Medicine, Tehran University of Medical Sciences, Tehran 1416634793, Iran
[9] Graduate School for Health Sciences, University of Bern, 3012 Bern, Switzerland
[10] Department of Cardiology, University Hospital of Bern, University of Bern, 3012 Bern, Switzerland
[11] Swiss Paraplegic Research, 6207 Nottwil, Switzerland

Contents

Search Strategy 3

Search strings........ 4

EMBASE 4

MEDLINE (Ovid) 6

Web of Science 8

Cochrane Central (Wiley)........ 10

Google Scholar 12

Effect of buckwheat products on a variety of cardiovascular risk markers........ 13

Meta-analysis summary 20

Table S4. Sensitivity analysis of the meta-analysis of randomized clinical trials comparing buckwheat supplementation versus control on blood lipids 21

Methodological quality assessment........ 24

Publication bias 25

Sensitivity analysis of the meta-analysis on total cholesterol after removing the non-grain component study 28

Table S5. Lipid profile markers at baseline........ 29

Leave-one-out analysis........ 30

Search Strategy

Table S1. Records identified in the search phase

Database searched	Source	Timespan covered	Records	Records after duplicates removed	Excluded records
EMBASE	Embase.com	1971 - 2022	830	820	10
Medline ALL	Ovid	1946 - 2022	593	129	464
Web of Science Core Collection*	Web of Knowledge	1975 - 2022	1419	842	577
Cochrane Central Register of Controlled Trials	Wiley	1992 - 2022	2	2	0
**Subtotal**			**2844**	**1793**	**1051**
Other sources: Google Scholar			200	144	56
**Total**			**3044**	**1937**	**1107**

Search strings

EMBASE

('lipid analysis'/exp OR (('marker'/exp/mj) AND ('lipid'/exp/mj)) OR 'cardiovascular risk factor'/de OR 'cardiovascular disease'/exp OR 'cardiometabolic disease'/de OR 'heart failure'/de OR 'congestive heart failure'/de OR 'heart disease'/de OR 'hypertensive heart disease'/de OR 'hypertensive heart failure'/de OR 'coronary artery disease'/de OR 'ischemic heart disease'/exp OR 'cerebrovascular accident'/de OR 'atherosclerotic cardiovascular disease'/de OR 'brain ischemia'/exp OR 'cardiovascular mortality'/de OR 'hypertension'/exp OR 'non insulin dependent diabetes mellitus'/exp OR 'insulin homeostasis'/de OR 'glucose homeostasis'/de OR 'insulin response'/exp OR 'glucose blood level'/exp OR 'insulin blood level'/exp OR 'hyperinsulinism'/exp OR 'hypoinsulinemia'/de OR 'impaired glucose tolerance'/de OR 'impaired fasting glucose'/de OR 'hyperglycemia'/de OR 'fasting glucose'/de OR 'glycosylated hemoglobin'/exp OR 'glycemic control'/de OR 'homa ir'/exp OR 'obesity'/exp OR 'body mass'/de OR 'body fat'/de OR 'adipose tissue'/exp OR 'waist circumference'/de OR 'waist hip ratio'/de OR 'peripheral circulation'/de OR 'abnormal blood pressure'/exp OR 'blood pressure'/exp OR (('vasoconstriction'/exp OR 'vasocongestion'/de OR 'vasodilator agent'/exp) AND ('enzyme activity'/de)) OR 'atherosclerosis'/de OR 'atherosclerotic plaque'/de OR 'carotid atherosclerosis'/de OR 'coronary artery atherosclerosis'/de OR 'arterial wall thickness'/de OR 'coronary artery calcification'/de OR 'endothelium'/exp OR 'endothelium cell'/exp OR 'endothelial function'/de OR 'endothelial dysfunction'/de OR 'inflammation'/exp OR 'oxidative stress'/de OR 'antioxidant'/exp OR 'body composition'/exp OR 'mortality'/exp OR 'nitric oxide'/de OR 'endothelium derived relaxing factor'/de OR 'endothelin 1'/de OR 'prostacyclin'/de OR 'intercellular adhesion molecule 1'/de OR 'vascular cell adhesion molecule 1'/de OR 'endothelial leukocyte adhesion molecule 1'/de OR 'PADGEM protein'/de OR 'arterial wall thickening'/de OR 'heart function'/exp OR 'pulse wave'/exp OR 'flow mediated dilatation'/de OR 'flow mediated dilation'/de OR 'peripheral arterial tonometry'/de OR 'echocardiography'/exp OR 'cardiovascular risk'/exp OR 'cardiometabolic risk'/de OR 'Metabolic Syndrome X'/de OR 'arterial stiffness'/de OR 'mortality'/exp OR 'cause of death'/de OR 'mortality risk'/de OR 'survival'/exp OR (hyperten* OR ((high) NEAR/3 (blood*) NEAR/3 (pressu*)) OR ((high) NEAR/3 (bloodpressu*)) OR ((cardiovascu* OR cardio-vascu* OR heart-diseas*) NEAR/3 (risk*)) OR ((cardiovascular OR cardio-vascular* OR coronar* OR cardia* OR heart OR myocard* OR cardiometab* OR cardio-metab* OR cardio-metabol*) NEAR/3 (accident* OR arrest* OR insuff* OR disease* OR event* OR infarct* OR disorder* OR function* OR dysfunct* OR disfunct* OR health* OR failure*)) OR cvd OR cvds OR cardiopath* OR cardio-path* OR angina OR ((ischemi* OR ischaemi* OR fail* OR attack* OR insufficien*) NEAR/3 (heart OR cardi* OR myocard*)) OR ((cerebrovascular*) NEAR/3 (accident* OR lesion* OR vasculopathy* OR vasculit* OR failure*)) OR cva OR stroke* OR ((brain OR cerebral OR cerebro* OR cerebrum*) NEAR/3 (ischemi* OR ischaemi* OR insult* OR accident* OR arrest* OR failure* OR accident*)) OR ((brain) NEAR/2 (attack*)) OR ((diabet* OR dm) NEAR/3 (type-2 OR type2 OR type-II OR non-insulin* OR noninsulin* OR adult-onset* OR slow-onset* OR maturity-onset*)) OR T2DM OR dmt2 OR dm2 OR T2-DM OR dm-t2 OR dm-2 OR niddm OR nid-dm OR MODY OR ((lipid* OR cholester* OR triacylglycerol* OR triglyceride* OR HDL* OR LDL* OR VLDL* OR VHDL*) NEAR/6 (blood* OR panel OR test* OR profile* OR serum OR plasma* OR concentration* OR level* OR peroxidat* OR autooxidat* OR autoxidat*)) OR ((glucose* OR sugar OR insulin*) NEAR/3 (level* OR blood OR serum OR plasma* OR concentration* OR toleran* OR sensitiv* OR insensitiv* OR resistan* OR response* OR dependen* OR homeosta* OR homoeosta*)) OR hypoglycemi* OR hypoglycaemi* OR hyperglycemi* OR hyperglycaemi* OR antihyperglycemi* OR prediabet* OR pre-diabet* OR fasting NEAR/2 glucose OR ((glycat* OR glyco*) NEAR/2 (hemoglob* OR haemoglob*)) OR HbA1c OR Hb-A1c OR hemoglobin-A1c OR haemoglobin-A1c OR HOMA-IR OR glycaem* OR glycem* OR glucosaemia

OR glucosemia OR hyperinsulin* OR hypoinsulin* OR insulinem* OR insulinaem* OR prediabet* OR pre-diabet* OR obes* OR over-weight* OR overweight OR adipos* OR 'body mass' OR BMI OR ((abdom* OR body) NEAR/3 (fat OR weight OR composition OR size)) OR ('weight gain*') OR ('weight loss*') OR (fat NEAR/3 (mass OR percentage* OR distribution)) OR (waist NEAR/3 (hip OR circumference*)) OR hypertensi* OR hypotensi* OR blood-pressure* OR ((vasocon* OR vasodil*) NEAR/12 (enzyme*)) OR ((endotheli*) NEAR/3 (vasorelax* OR relax* OR sensit* OR constrict* OR contract* OR adhes*)) OR endothelin-1 OR prostaglandin-i2 OR PGI2 OR intercellular-adhesion-molecule-1 OR ICAM-1 OR CD54-antigen* OR CD-54-antigen* OR nitric-oxid* OR genosyl OR nitrogen-mono* OR noxivent OR vascular-cell-adhesion-molecule-1 OR VCAM-1 OR CD106-antigen* OR CD-106-antigen* OR endothelial-leukocyte-adhesion-molecule-1 OR e-selectin OR ELAM-1 OR selectin-e OR PADGEM-protein OR P-selectin OR selectin-P OR ((vascular OR aort* OR arter*) NEAR/2 (stiff* OR wall OR thick*)) OR ((ventricular OR ventricle OR heart) NEAR/3 (funct* OR dysfunct* OR rate)) OR ventricle-stroke-volum* OR pulse-wave* OR ((arterial* OR artery) NEAR/3 (wave* OR pulse*)) OR flow-mediated-dilat* OR peripheral-arterial-tonometr* OR echocardiograph* OR cardioechograph* OR ((cardiac OR cardial OR heart) NEAR/3 (echograp* OR scan* OR echocard*)) OR ((echo) NEAR/3 (cardiog*)) OR ((cardiovascular* OR cardio-vasc* OR cardiac-vasc* OR cardiometabol* OR cardio-metabol* OR metabol*) NEAR/3 (risk* OR syndrome*)) OR CV-risk OR hypercholesterol* OR atheroscler* OR athero-scler* OR ((arteriosclerotic* OR arterio-sclerotic*) NEAR/2 (plaque*)) OR ((intima-media OR intimamedia) NEAR/3 thickness*) OR ((arterial) NEAR/1 (wall* OR thickness*)) OR ((coronar*) NEAR/3 (calcif*)) OR endothel* OR antioxid* OR anti-oxid* OR inflamma* OR ((oxidat*) NEAR/3 (stress* OR damage* OR injur*)) OR ((body OR bodies) NEAR/3 (compos* OR adipos* OR distribut* OR fat OR water OR cholest* OR potas*)) OR ((reactive) NEAR/3 (oxygen*) NEAR/3 (metabolite* OR species)) OR lipoperoxidat* OR lipo-peroxidat* OR isoprostan* OR malonaldehyde* OR lipoxygenase* OR myeloperoxidase* OR mortalit* OR death* OR fatalit* OR survival* OR die OR died OR passed-away* OR dead):ab,ti,kw) **AND** ('fagopyrum'/exp OR (buckwheat* OR buck-wheat* OR fagopyrumesculent* OR ((fagopyrum* OR Polygonum*) NEAR/3 (escul* OR tatar* OR tarar* OR tartar* OR spp OR tartica*)) OR ((f) NEAR/2 (esculentum OR tataricum OR tartar OR tartarian OR tarticarium))):ab,ti,kw)

MEDLINE (Ovid)

(((exp *"Biomarkers"/) AND (exp *"Lipids"/)) OR exp "Heart Disease Risk Factors"/ OR exp "Cardiovascular Diseases"/ OR exp "Coronary Artery Disease"/ OR exp "Stroke"/ OR exp "Diabetes Mellitus, Type 2"/ OR exp "Hyperinsulinism"/ OR exp "Hyperglycemia"/ OR "Glycemic Control"/ OR exp "Overweight"/ OR exp "Adipose Tissue"/ OR exp "Waist Circumference"/ OR "Waist-Hip Ratio"/ OR exp "Blood Pressure"/ OR (("Vasoconstriction"/ OR exp "Vasodilator Agents"/) AND (en.fs)) OR exp "Atherosclerosis"/ OR "Plaque, Atherosclerotic"/ OR exp "Endothelium"/ OR exp "Inflammation"/ OR exp "Oxidative Stress"/ OR exp "Antioxidants"/ OR exp "Body Composition"/ OR "Nitric Oxide"/ OR ' "Endothelium-Dependent Relaxing Factors"/ OR "Endothelin-1"/ OR "Intercellular Adhesion Molecule-1"/ OR "Vascular Cell Adhesion Molecule-1"/ OR "E-Selectin"/ OR "P-Selectin"/ OR "Pulse"/ OR exp "Pulse Wave Analysis"/ OR exp "Echocardiography"/ OR "Vascular Stiffness"/ OR exp "Mortality"/ OR mo.fs OR "Survival"/ OR (hyperten* OR ((high) ADJ3 (blood*) ADJ3 (pressu*)) OR ((high) ADJ3 (bloodpressu*)) OR ((cardiovascu* OR cardio-vascu* OR heart-diseas*) ADJ3 (risk*)) OR ((cardiovascular OR cardio-vascular* OR coronar* OR cardia* OR heart OR myocard* OR cardiometab* OR cardio-metab* OR cardio-metabol*) ADJ3 (accident* OR arrest* OR insuff* OR disease* OR event* OR infarct* OR disorder* OR function* OR dysfunct* OR disfunct* OR health* OR failure*)) OR cvd OR cvds OR cardiopath* OR cardio-path* OR angina OR ((ischemi* OR ischaemi* OR fail* OR attack* OR insufficien*) ADJ3 (heart OR cardi* OR myocard*)) OR ((cerebrovascular*) ADJ3 (accident* OR lesion* OR vasculopathy* OR vasculit* OR failure*)) OR cva OR stroke* OR ((brain OR cerebral OR cerebro* OR cerebrum*) ADJ3 (ischemi* OR ischaemi* OR insult* OR accident* OR arrest* OR failure* OR accident*)) OR ((brain) ADJ2 (attack*)) OR ((diabet* OR dm) ADJ3 (type-2 OR type2 OR type-II OR non-insulin* OR noninsulin* OR adult-onset* OR slow-onset* OR maturity-onset*)) OR T2DM OR dmt2 OR dm2 OR T2-DM OR dm-t2 OR dm-2 OR niddm OR nid-dm OR MODY OR ((lipid* OR cholester* OR triacylglycerol* OR triglyceride* OR HDL* OR LDL* OR VLDL* OR VHDL*) ADJ6 (blood* OR panel OR test* OR profile* OR serum OR plasma* OR concentration* OR level* OR peroxidat* OR autooxidat* OR autoxidat*)) OR ((glucose* OR sugar OR insulin*) ADJ3 (level* OR blood OR serum OR plasma* OR concentration* OR toleran* OR sensitiv* OR insensitiv* OR resistan* OR response* OR dependen* OR homeosta* OR homoeosta*)) OR hypoglycemi* OR hypoglycaemi* OR hyperglycemi* OR hyperglycaemi* OR antihyperglycemi* OR prediabet* OR pre-diabet* OR fasting ADJ2 glucose OR ((glycat* OR glyco*) ADJ2 (hemoglob* OR haemoglob*)) OR HbA1c OR Hb-A1c OR hemoglobin-A1c OR haemoglobin-A1c OR HOMA-IR OR glycaem* OR glycem* OR glucosaemia OR glucosemia OR hyperinsulin* OR hypoinsulin* OR insulinem* OR insulinaem* OR prediabet* OR pre-diabet* OR obes* OR over-weight* OR overweight OR adipos* OR 'body mass' OR BMI OR ((abdom* OR body) ADJ3 (fat OR weight OR composition OR size)) OR ('weight gain*') OR ('weight loss*') OR (fat ADJ3 (mass OR percentage* OR distribution)) OR (waist ADJ3 (hip OR circumference*)) OR hypertensi* OR hypotensi* OR blood-pressure* OR ((vasocon* OR vasodil*) ADJ12 (enzyme*)) OR ((endotheli*) ADJ3 (vasorelax* OR relax* OR sensit* OR constrict* OR contract* OR adhes*)) OR endothelin-1 OR prostaglandin-i2 OR PGI2 OR intercellular-adhesion-molecule-1 OR ICAM-1 OR CD54-antigen* OR CD-54-antigen* OR nitric-oxid* OR genosyl OR nitrogen-mono* OR noxivent OR vascular-cell-adhesion-molecule-1 OR VCAM-1 OR CD106-antigen* OR CD-106-antigen* OR endothelial-leukocyte-adhesion-molecule-1 OR e-selectin OR ELAM-1 OR selectin-e OR PADGEM-protein OR P-selectin OR selectin-P OR ((vascular OR aort* OR arter*) ADJ2 (stiff* OR wall OR thick*)) OR ((ventricular OR ventricle OR heart) ADJ3 (funct* OR dysfunct* OR rate)) OR ventricle-stroke-volum* OR pulse-wave* OR ((arterial* OR artery) ADJ3 (wave* OR pulse*)) OR flow-mediated-dilat* OR peripheral-arterial-tonometr* OR echocardiograph* OR cardioechograph* OR ((cardiac OR cardial OR heart) ADJ3 (echograp* OR scan* OR echocard*)) OR ((echo) ADJ3 (cardiog*)) OR ((cardiovascular* OR cardio-vasc* OR cardiac-vasc* OR cardiometabol* OR cardio-metabol* OR metabol*) ADJ3 (risk* OR syndrome*)) OR CV-risk OR

hypercholesterol* OR atheroscler* OR athero-scler* OR ((arteriosclerotic* OR arterio-sclerotic*) ADJ2 (plaque*)) OR ((intima-media OR intimamedia) ADJ3 thickness*) OR ((arterial) ADJ1 (wall* OR thickness*)) OR ((coronar*) ADJ3 (calcif*)) OR endothel* OR antioxid* OR anti-oxid* OR inflamma* OR ((oxidat*) ADJ3 (stress* OR damage* OR injur*)) OR ((body OR bodies) ADJ3 (compos* OR adipos* OR distribut* OR fat OR water OR cholest* OR potas*)) OR ((reactive) ADJ3 (oxygen*) ADJ3 (metabolite* OR species)) OR lipoperoxidat* OR lipo-peroxidat* OR isoprostan* OR malonaldehyde* OR lipoxygenase* OR myeloperoxidase* OR mortalit* OR death* OR fatalit* OR survival* OR die OR died OR passed-away* OR dead).ab,ti,kf.) **AND** ("Fagopyrum"/ OR (buckwheat* OR buck-wheat* OR fagopyrumesculent* OR ((fagopyrum* OR Polygonum*) ADJ3 (escul* OR tatar* OR tarar* OR tartar* OR spp OR tartica*)) OR ((f) ADJ2 (esculentum OR tataricum OR tartar OR tartarian OR tarticarium))).ab,ti,kf.)

Web of Science

TS=(((hyperten* OR ((high) NEAR/2 (blood*) NEAR/2 (pressu*)) OR ((high) NEAR/2 (bloodpressu*)) OR ((cardiovascu* OR cardio-vascu* OR heart-diseas*) NEAR/2 (risk*)) OR ((cardiovascular OR cardio-vascular* OR coronar* OR cardia* OR heart OR myocard* OR cardiometab* OR cardio-metab* OR cardio-metabol*) NEAR/2 (accident* OR arrest* OR insuff* OR disease* OR event* OR infarct* OR disorder* OR function* OR dysfunct* OR disfunct* OR health* OR failure*)) OR cvd OR cvds OR cardiopath* OR cardio-path* OR angina OR ((ischemi* OR ischaemi* OR fail* OR attack* OR insufficien*) NEAR/2 (heart OR cardi* OR myocard*)) OR ((cerebrovascular*) NEAR/2 (accident* OR lesion* OR vasculopathy* OR vasculit* OR failure*)) OR cva OR stroke* OR ((brain OR cerebral OR cerebro* OR cerebrum*) NEAR/2 (ischemi* OR ischaemi* OR insult* OR accident* OR arrest* OR failure* OR accident*)) OR ((brain) NEAR/2 (attack*)) OR ((diabet* OR dm) NEAR/2 (type-2 OR type2 OR type-II OR non-insulin* OR noninsulin* OR adult-onset* OR slow-onset* OR maturity-onset*)) OR T2DM OR dmt2 OR dm2 OR T2-DM OR dm-t2 OR dm-2 OR niddm OR nid-dm OR MODY OR ((lipid* OR cholester* OR triacylglycerol* OR triglyceride* OR HDL* OR LDL* OR VLDL* OR VHDL*) NEAR/5 (blood* OR panel OR test* OR profile* OR serum OR plasma* OR concentration* OR level* OR peroxidat* OR autooxidat* OR autoxidat*)) OR ((glucose* OR sugar OR insulin*) NEAR/2 (level* OR blood OR serum OR plasma* OR concentration* OR toleran* OR sensitiv* OR insensitiv* OR resistan* OR response* OR dependen* OR homeosta* OR homoeosta*)) OR hypoglycemi* OR hypoglycaemi* OR hyperglycemi* OR hyperglycaemi* OR antihyperglycemi* OR prediabet* OR pre-diabet* OR fasting NEAR/2 glucose OR ((glycat* OR glyco*) NEAR/2 (hemoglob* OR haemoglob*)) OR HbA1c OR Hb-A1c OR hemoglobin-A1c OR haemoglobin-A1c OR HOMA-IR OR glycaem* OR glycem* OR glucosaemia OR glucosemia OR hyperinsulin* OR hypoinsulin* OR insulinem* OR insulinaem* OR prediabet* OR pre-diabet* OR obes* OR over-weight* OR overweight OR adipos* OR "body mass" OR BMI OR ((abdom* OR body) NEAR/2 (fat OR weight OR composition OR size)) OR ("weight gain*") OR ("weight loss*") OR (fat NEAR/2 (mass OR percentage* OR distribution)) OR (waist NEAR/2 (hip OR circumference*)) OR hypertensi* OR hypotensi* OR blood-pressure* OR ((vasocon* OR vasodil*) NEAR/12 (enzyme*)) OR ((endotheli*) NEAR/2 (vasorelax* OR relax* OR sensit* OR constrict* OR contract* OR adhes*)) OR endothelin-1 OR prostaglandin-i2 OR PGI2 OR intercellular-adhesion-molecule-1 OR ICAM-1 OR CD54-antigen* OR CD-54-antigen* OR nitric-oxid* OR genosyl OR nitrogen-mono* OR noxivent OR vascular-cell-adhesion-molecule-1 OR VCAM-1 OR CD106-antigen* OR CD-106-antigen* OR endothelial-leukocyte-adhesion-molecule-1 OR e-selectin OR ELAM-1 OR selectin-e OR PADGEM-protein OR P-selectin OR selectin-P OR ((vascular OR aort* OR arter*) NEAR/2 (stiff* OR wall OR thick*)) OR ((ventricular OR ventricle OR heart) NEAR/2 (funct* OR dysfunct* OR rate)) OR ventricle-stroke-volum* OR pulse-wave* OR ((arterial* OR artery) NEAR/2 (wave* OR pulse*)) OR flow-mediated-dilat* OR peripheral-arterial-tonometr* OR echocardiograph* OR cardioechograph* OR ((cardiac OR cardial OR heart) NEAR/2 (echograp* OR scan* OR echocard*)) OR ((echo) NEAR/2 (cardiog*)) OR ((cardiovascular* OR cardio-vasc* OR cardiac-vasc* OR cardiometabol* OR cardio-metabol* OR metabol*) NEAR/2 (risk* OR syndrome*)) OR CV-risk OR hypercholesterol* OR atheroscler* OR athero-scler* OR ((arteriosclerotic* OR arterio-sclerotic*) NEAR/2 (plaque*)) OR ((intima-media OR intimamedia) NEAR/2 thickness*) OR ((arterial) NEAR/1 (wall* OR thickness*)) OR ((coronar*) NEAR/2 (calcif*)) OR endothel* OR antioxid* OR anti-oxid* OR inflamma* OR ((oxidat*) NEAR/2 (stress* OR damage* OR injur*)) OR ((body OR bodies) NEAR/2 (compos* OR adipos* OR distribut* OR fat OR water OR cholest* OR potas*)) OR ((reactive) NEAR/2 (oxygen*) NEAR/2 (metabolite* OR species)) OR lipoperoxidat* OR lipo-peroxidat* OR isoprostan* OR malonaldehyde* OR lipoxygenase* OR myeloperoxidase* OR mortalit* OR death* OR fatalit* OR survival* OR die OR died OR passed-away* OR dead)) **AND** ((buckwheat* OR buck-wheat* OR fagopyrumesculent* OR

((fagopyrum* OR Polygonum*) NEAR/2 (escul* OR tatar* OR tarar* OR tartar* OR spp OR tartica*)) OR ((f) NEAR/2 (esculentum OR tataricum OR tartar OR tartarian OR tarticarium)))))

Cochrane Central (Wiley)

((hyperten* OR ((high) NEAR/3 (blood*) NEAR/3 (pressu*)) OR ((high) NEAR/3 (bloodpressu*)) OR ((cardiovascu* OR cardio NEXT vascu* OR heart NEXT diseas*) NEAR/3 (risk*)) OR ((cardiovascular OR cardio NEXT vascular* OR coronar* OR cardia* OR heart OR myocard* OR cardiometab* OR cardio NEXT metab* OR cardio NEXT metabol*) NEAR/3 (accident* OR arrest* OR insuff* OR disease* OR event* OR infarct* OR disorder* OR function* OR dysfunct* OR disfunct* OR health* OR failure*)) OR cvd OR cvds OR cardiopath* OR cardio NEXT path* OR angina OR ((ischemi* OR ischaemi* OR fail* OR attack* OR insufficien*) NEAR/3 (heart OR cardi* OR myocard*)) OR ((cerebrovascular*) NEAR/3 (accident* OR lesion* OR vasculopathy* OR vasculit* OR failure*)) OR cva OR stroke* OR ((brain OR cerebral OR cerebro* OR cerebrum*) NEAR/3 (ischemi* OR ischaemi* OR insult* OR accident* OR arrest* OR failure* OR accident*)) OR ((brain) NEAR/2 (attack*)) OR ((diabet* OR dm) NEAR/3 (type NEXT 2 OR type2 OR type NEXT II OR non NEXT insulin* OR noninsulin* OR adult NEXT onset* OR slow NEXT onset* OR maturity NEXT onset*)) OR T2DM OR dmt2 OR dm2 OR T2 NEXT DM OR dm NEXT t2 OR dm NEXT 2 OR niddm OR nid NEXT dm OR MODY OR ((lipid* OR cholester* OR triacylglycerol* OR triglyceride* OR HDL* OR LDL* OR VLDL* OR VHDL*) NEAR/6 (blood* OR panel OR test* OR profile* OR serum OR plasma* OR concentration* OR level* OR peroxidat* OR autooxidat* OR autoxidat*)) OR ((glucose* OR sugar OR insulin*) NEAR/3 (level* OR blood OR serum OR plasma* OR concentration* OR toleran* OR sensitiv* OR insensitiv* OR resistan* OR response* OR dependen* OR homeosta* OR homoeosta*)) OR hypoglycemi* OR hypoglycaemi* OR hyperglycemi* OR hyperglycaemi* OR antihyperglycemi* OR prediabet* OR pre NEXT diabet* OR fasting NEAR/2 glucose OR ((glycat* OR glyco*) NEAR/2 (hemoglob* OR haemoglob*)) OR HbA1c OR Hb NEXT A1c OR hemoglobin NEXT A1c OR haemoglobin NEXT A1c OR HOMA NEXT IR OR glycaem* OR glycem* OR glucosaemia OR glucosemia OR hyperinsulin* OR hypoinsulin* OR insulinem* OR insulinaem* OR prediabet* OR pre NEXT diabet* OR obes* OR over NEXT weight* OR overweight OR adipos* OR 'body mass' OR BMI OR ((abdom* OR body) NEAR/3 (fat OR weight OR composition OR size)) OR ('weight gain*') OR ('weight loss*') OR (fat NEAR/3 (mass OR percentage* OR distribution)) OR (waist NEAR/3 (hip OR circumference*)) OR hypertensi* OR hypotensi* OR blood NEXT pressure* OR ((vasocon* OR vasodil*) NEAR/12 (enzyme*)) OR ((endotheli*) NEAR/3 (vasorelax* OR relax* OR sensit* OR constrict* OR contract* OR adhes*)) OR endothelin NEXT 1 OR prostaglandin NEXT i2 OR PGI2 OR intercellular NEXT adhesion NEXT molecule NEXT 1 OR ICAM NEXT 1 OR CD54 NEXT antigen* OR CD NEXT 54 NEXT antigen* OR nitric NEXT oxid* OR genosyl OR nitrogen NEXT mono* OR noxivent OR vascular NEXT cell NEXT adhesion NEXT molecule NEXT 1 OR VCAM NEXT 1 OR CD106 NEXT antigen* OR CD NEXT 106 NEXT antigen* OR endothelial NEXT leukocyte NEXT adhesion NEXT molecule NEXT 1 OR e NEXT selectin OR ELAM NEXT 1 OR selectin NEXT e OR PADGEM NEXT protein OR P NEXT selectin OR selectin NEXT P OR ((vascular OR aort* OR arter*) NEAR/2 (stiff* OR wall OR thick*)) OR ((ventricular OR ventricle OR heart) NEAR/3 (funct* OR dysfunct* OR rate)) OR ventricle NEXT stroke NEXT volum* OR pulse NEXT wave* OR ((arterial* OR artery) NEAR/3 (wave* OR pulse*)) OR flow NEXT mediated NEXT dilat* OR peripheral NEXT arterial NEXT tonometr* OR echocardiograph* OR cardioechograph* OR ((cardiac OR cardial OR heart) NEAR/3 (echograp* OR scan* OR echocard*)) OR ((echo) NEAR/3 (cardiog*)) OR ((cardiovascular* OR cardio NEXT vasc* OR cardiac NEXT vasc* OR cardiometabol* OR cardio NEXT metabol* OR metabol*) NEAR/3 (risk* OR syndrome*)) OR CV NEXT risk OR hypercholesterol* OR atheroscler* OR athero NEXT scler* OR ((arteriosclerotic* OR arterio NEXT sclerotic*) NEAR/2 (plaque*)) OR ((intima NEXT media OR intimamedia) NEAR/3 thickness*) OR ((arterial) NEAR/1 (wall* OR thickness*)) OR ((coronar*) NEAR/3 (calcif*)) OR endothel* OR antioxid* OR anti NEXT oxid* OR inflamma* OR ((oxidat*) NEAR/3 (stress* OR damage* OR injur*)) OR ((body OR bodies) NEAR/3 (compos* OR adipos* OR distribut* OR fat OR water OR cholest* OR potas*)) OR ((reactive) NEAR/3 (oxygen*) NEAR/3 (metabolite* OR species)) OR lipoperoxidat* OR lipo

NEXT peroxidat* OR isoprostan* OR malonaldehyde* OR lipoxygenase* OR myeloperoxidase* OR mortalit* OR death* OR fatalit* OR survival* OR die OR died OR passed NEXT away* OR dead):ab,ti,kw) **AND** ((buckwheat* OR buck NEXT wheat* OR fagopyrumesculent* OR ((fagopyrum* OR Polygonum*) NEAR/3 (escul* OR tatar* OR tarar* OR tartar* OR spp OR tartica*)) OR ((f) NEAR/2 (esculentum OR tataricum OR tartar OR tartarian OR tarticarium))):ab,ti,kw)

Google Scholar

Buckwheat | buck-wheat | fagopyrumesculent | "fagopyrum esculentum" cardiovascular | cardiometabolic | diabetes | lipid | glucose | obesity | "blood pressure" | Atherosclerosis | inflammation | "oxidative stress" | moratlity | survival | insulin | BMI | "body fat | mass"

Effect of buckwheat products on a variety of cardiovascular risk markers

Table S2. Human interventional and randomized controlled studies describing the effect of buckwheat intake on CVD risk markers

Study (year) [ref.]	Intervention (N)/control (n)	Duration (weeks)	Intervention type	Intervention dose	Control	Health Status	Age (years)*	CVD risk marker	Effect in intervention arm	Difference between arms (95%CI) †
Archimowicz-Cyrylowska et al. (1996) [1]	20/40	12	BW herb mixture	*Ad libitum*	Troxerutin and Ruscus mixture	T2D	20-75	Glucose	↓↓	-0.35(-1.54;0.84)
								HbA1c	↓↓	**0.58(0.45;0.70)**
								HDL	↑↑	0.14(-0.33;0.62)
								TC	↓↓	0.30(-0.20;0.80)
								TG	↓↓	0.19(-0.08;0.46)
Bijlani (1984) [2]	7/5	4	Sieved BW flour	100 g	Lunch cereal intake	Healthy	19-22	HDL	↑↑	**-0.28(-0.51;-0.06)**
								LDL	↑	-0.05(-0.28;0.18)
								TC	↑	-0.13(-0.73;0.48)
								Glucose	↑	-0.05(-0.13;0.02)
								vLDL	↓	-0.20(-0.49;0.09)
Bijlani (1985) [3]	8/9	12	Whole BW flour	100 g	Breakfast cereal	Healthy	19-34	Glucose	↓↓	**15.5(10.9;20.1)**
								HDL	↑↑	0.003(-0.17;0.18)
								LDL	↑	**-0.83(-1.34;-0.32)**
								TC	↓	-0.06(-0.53;0.41)
								TG	↓	**0.31(0.11;0.502)**
								vLDL	↓	**-1.39(-0.94;-0.19)**
Dinu et al. (2017) [4]	10/9	6	BW-enriched foods	80 g of 100% BW pasta and BW-rich products: 60 g of tacks, 40 g biscuits and 50 g of flakes (daily)	Usual gluten-free diet	Gluten sensitivity	44	Glucose	↑	**0.33(0.26;0.40)**
								HDL	↓	**0.13(0.07;0.19)**
								IFN-γ	↓	-4.80(-15.51;5.91)
								IL-10	↑	2.40(-0.72;5.52)
								IL-12	↓	**-1.50(-2.33;-0.67)**
								IL-1ra	↑	-2.50(-6.04;1.04)
								IL-4	↓	**-0.03(-0.06;0.004)**
								IL-6	↓	-0.70 (-2.03;0.63)

Study (year) [ref.]	Intervention (N)/control (n)	Duration (weeks)	Intervention type	Intervention dose	Control	Health Status	Age (years)*	CVD risk marker	Effect in intervention arm	Difference between arms (95%CI) †
								IL-8	↓	7.50(-4.11;19.11)
								LDL	↑	0.00(-0.76;0.76)
								MCP-1	↓↓	-11.10(-23.56;1.36)
								TNFα	↓	0.10(-0.33;0.53)
								TC	↓	**0.26(0.15;0.36)**
								TG	↓	-0.09(-0.19;0.01)
								VEGF	↓	-0.70(-47.36;45.96)
Huang et al. (2009) [5]	35/35	8	Tatary BW mixture	Not stated	Control drug	T2D	53	Glucose	↓↓	**-2.69(-3.14;-2.24)**
								HbA1c	↓↓	**-2.15(-2.24;-2.06)**
								HDL	↓↓	**-1.91(-1.95;-1.87)**
								LDL	↓↓	**-0.70(-0.73;-0.67)**
								TC	↓↓	**-1.22(-1.29;-1.15)**
								TG	↓↓	**-1.68(-1.80;-1.56)**
Mišan et al. (2017) [6]	12/22	5	BW-enriched instant porridge	80 g	80g maize instant porridge	HC subjects	56	Adiponectin	↑↑	**4.10(3.13;5.07)**
								CRP	↓	**-0.80(-1.16;-0.439**
								Fat mass	↓	-0.15(-2.91;2.61)
								Glucose	↓	-0.04(-0.22;0.15)
								HDL	↑↑	-0.07(-0.18;0.04)
								LDL	↓↓	0.42(0.17;0.67)
								TC	↓↓	0.49(0.14;0.85)
								TG	↓↓	0.13(-0.10;0.36)
								Weight	↑	0.10(-0.32;0.52)
Nishimura et al. (2016) [7]	73/71	12	Rutin-rich Tartary BW foods	Tartary BW noodles (80g) and cookies (50g)	Wheat-based noodles (80g) and cookies (50g)	Healthy	54.1	Atherogenic index	↓	**0.05(0.05;0.06)**
								BMI	↓	0.10(-0.56;0.76)
								BF	↓	-0.26(-0.31;-0.21)
								DBP	↑	0.15(-2.26;2.56)

Study (year) [ref.]	Intervention (N)/control (n)	Duration (weeks)	Intervention type	Intervention dose	Control	Health Status	Age (years)*	CVD risk marker	Effect in intervention arm	Difference between arms (95%CI) †
								HDL	↓	0.04(-0.05;0.13)
								LDL	↓	0.05(-0.19;0.29)
								oxLDL	↓	**-2.160(-4.31;-0.01)**
								SBP	↓	0.98(-2.23;4.19)
								TBARS	↑	**-0.98(-0.76;-0.68)**
								TC	↓	-0.02(-0.21;0.17)
								TG	↑	-0.12(-0.24;0.01)
								Weight	↓	0.24(-1.97;2.45)
Qiu et al. (2016) (a) [8]	52/52	4	Tartary BW foods	Not stated	Diet plan/education	T2D	58.8	DBP	↓	-1.23(-3.91;1.45)
								SBP	↑	2.35(-1.15;5.85)
Qiu et al. (2016) (b) [9]	80/85	4	Tartary BW foods (e.g., kernel, noodle, powder)	*Ad libitum*	Refined grains	T2D	56.9	BMI	↓	0.01(-0.61;0.63)
								Glucose	↓	0.35(-0.20;0.90)
								HbA1c	↓	**-0.14(-0.17;-0.11)**
								HDL	↓	0.01(-0.04;0.06)
								HOMA-IR	↓	**-0.40;-0.60;-0.21)**
								Insulin	↓↓	**-1.29(-1.67;-0.91)**
								LDL	↓↓	**0.21(0.04;0.38)**
								TC	↓↓	0.21(-0.01;0.43)
								TG	↓	-0.06(-0.35;0.23)
								Weight	↓	0.11(-1.78;2.00)
Shakib et al. (2011)[10]	20/NA	6	BW-yoghurt mixture	30 g BW seeds and 160 g yoghurt	No control	HC and NIDDM patients	30-50	Glucose	↓↓	**5.55(5.13;5.96)**
								HDL	↑↑	**-0.09(-0.10;-0.08)**
								LDL	↓↓	**0.61(0.58;0.64)**
								TC	↓↓	1.**87(1.85;1.89)**
								TG	↓↓	**0.82(0.81;0.83)**
Sofi et al. (2016) [11]	10/11	8	BW-enriched foods	500 g /week of pasta, 1 kg/week		At risk for CVDs	51.3	Glucose	↓↓	0.10(-0.18;0.38)
								HDL	↓↓	0.08(-0.27;0.43)

Study (year) [ref.]	Intervention (N)/control (n)	Duration (weeks)	Intervention type	Intervention dose	Control	Health Status	Age (years)*	CVD risk marker	Effect in intervention arm	Difference between arms (95%CI) †
				of bread, 500 g/month of crackers and 1 kg/month of biscuits				HOMA-IR	↓↓	-0.50(-0.28;3.48)
								IL-10	↓	2.90(-1.06;6.86)
								IL-12	↓	1.60(-0.28;3.48)
								IL-17	↓	**0.50(0.356;0.64)**
								IL-1ra	↓	5.90(-27.29;39.09)
								IL-4	*No effect*	0.1(0.1;0.1)
								IL-6	*No effect*	**0.90(0.50;1.30)**
								IL-8	↓	-14.90(-35.38;5.58)
								Insulin	↓	**-2.20(-3.98;-0.42)**
								IP-10	↓	-107.2(-226.9;12.50)
								LDL	↓↓	0.35(-0.23; 0.93)
					Wheat-based bread, pasta, biscuits and crackers			MIP-1α	↓	-7(-27.65;13.65)
								Malondialdehyde	↓↓	**-12.7(-21.29;-4.11)**
								MCP-1	↓↓	-0.70(-6.76;5.36)
								ROS-granulocytes	↑	**-184(-251;-116)**
								ROS-lymphocytes	↓	**-205(-238;-172)**
								TNFα	↑	3.1(0.04;6.16)
								Antioxidant capac.	↑↑	1.6(1.42;1.78)
								TC	↓↓	0.32(-0.348;0.98)
								TG	↓↓	0.17(-0.25;0.58)
								VEGF	↓	3.3(-0.08;6.68)
Stokić et al. (2015) [12]	20/20	4			300 g wheat bread		59.5	BMI	*No effect*	-0.10(-0.41;0.21)

Study (year) [ref.]	Intervention (N)/control (n)	Duration (weeks)	Intervention type	Intervention dose	Control	Health Status	Age (years)*	CVD risk marker	Effect in intervention arm	Difference between arms (95%CI) †
			BW-enriched wheat bread	50 g wholegrain BW flour		At risk for CVD		CRP	↓↓	**0.12(0.03;0.21)**
								DBP	↓↓	1.25(-0.25;2.75)
								HDL	↑	-0.03(-0.13;0.07)
								LDL	↓↓	**0.59(0.41;0.77)**
								SBP	↓↓	**2(0.28;3.72)**
								TC	↓	**0.29(0.07;0.51)**
								TG	↑	-0.04(-0.16;0.08)
Stringer et al. (2013) [13]	7/6	1	BW crackers	50 g wholegrain BW flour	Rice cracker	Healthy and T2D	49.5	Apo A1	↑↑	**0.45(0.19;0.71)**
								Apo B	↓	**0.02(0.019;0.03)**
								Apo C3	↑	**0.06(0.05;0.06)**
								Glucose	↑↑	**0.26(0.03;0.49)**
								HDL	↓	0.03(-0.06;0.12)
								LDL	↑	-0.17(-0.38;0.04)
								TC	↑	-0.23(-0.49;0.03)
								TG	↑	-0.19(-0.36;-0.02)
Wieslander (2011) [14]	30/32	4	BW cookies	100 g (rutin≈360 mg)	Common BW cookies (rutin≈17 mg)	Healthy	46	HDL	↓↓	**0.21(0.13;0.29)**
								HsCRP	↓	0.25(-0.55;1.05)
								ECP	↓	0.10(-2.66;2.86)
								Myeloperoxidase	↓	13(-12.55;38.55)
								sPLA2-IIA	↓	0.50(-0.33;1.33)
								TC	↓↓	**0.72(0.43;1.01)**
Zhao and Guan (2003) [15]	60/NA	8	BW flour	Not stated	No control	T2D	26-67	DBP	↓	2(-0.82;4.82)
								Glucose	↓↓	**1.2(0.78;1.62)**
								HDL	↑↑	**-0.1(-0.18;-0.02)**
								LDL	↓	0.1(-0.09;0.29)
								SBP	↓↓	**6(1.66;10.34)**

Study (year) [ref.]	Intervention (N)/control (n)	Duration (weeks)	Intervention type	Intervention dose	Control	Health Status	Age (years)*	CVD risk marker	Effect in intervention arm	Difference between arms (95%CI) †
								TC	↓↓	**0.3(0.11;0.49)**
								TG	↓	**0.2(0;0.4)**
								Weight	*No effect*	-
Zheng et al. (1991) [16]	19/NA	12	Tartary BW flour	50g	No control	NIDDM	53.8	Apo A1	↑	**-14.32(-17.29;-11.35)**
								Apo B	↓↓	**20.31(17.22;23.40)**
								Glucose	↓	**0.24(0.01;0.47)**
								HbA1c	↑	**-8.25(-10.02;-6.48)**
								Insulin	↑	**-42(-44;-41)**
								TC	↓	**0.66(0.57;0.75)**
								TG	↓↓	**0.65(0.49;0.81)**

** Age is indicated mean age and in case mean was not available, age range is provided.*

† Data are provided as difference of the mean differences in the control/intervention group accompanied by their respective 95% confidence intervals. In all cases that this difference was not possible/applicable to quantify this difference, the mean difference in the intervention arm (accompanied by 95%CI) is provided in grey shading – statistically significant values are bolded.

Note: ↑: increase; ↓: decrease; ↑↑: statistically significant increase; ↓↓: statistically significant decrease. Abbreviations: HC: patients with hypercholesterolemia; T2D: type 2 diabetes; NCGS: Non-celiac gluten sensitivity; NIDDM: Non-insulin-dependent diabetes mellitus TC: Total cholesterol; TG: triglycerides; BMI: Body mass index; CRP: C-reactive protein; SBP: systolic blood pressure; DBP: diastolic blood pressure; TBARS: Thiobarbituric acid reactive substance; ECP: Serum eosinophilic cation protein; sPLA2-IIA: sPLA2 - group IIA secretory phospholipase A2; MPO Serum myeloperoxidase; HsCRP: high-sensitivity C-reactive protein; VEGF: Vascular endothelial growth factor; Apo A1: Apolipoprotein A1; Apo A2: Apolipoprotein A2; Apo B: Apolipoprotein B; Apo C3: Apolipoprotein C3; ROS: reactive oxygen species; MIP-1α: Macrophage Inflammatory Protein-1 beta; IP-10: Interferon Gamma-Induced Protein 10; HOMA-IR: Homeostatic Model Assessment for Insulin Resistance; oxLDL: oxidized LDL cholesterol; HbA1c: Glycated hemoglobin; HDL: High density lipoprotein; LDL: Low density lipoprotein; BF: Body fatness; AIP: Atherogenic index of plasma; TNF-α: Tumor necrosis factor; vLDL: very low density lipoprotein; All 'IL-' abbreviations followed by a number, indicate 'Interleukin': in case of IL-1ra, the 'ra' indicates 'receptor agonist'; Trial duration indicates only the first period in case of crossover trials.

[1] B. Archimowicz-Cyrylowska, B. Adamek, M. Droździk, L. Samochowiec, and J. Wójcicki. Clinical effect of buckwheat herb, Ruscus extract and troxerutin on retinopathy and lipids in diabetic patients. Phytother Res 10 (1996) 659-662.
[2] R.L. Bijlani, S. Sud, A. Sahi, B.M. Gandhi, and B.N. Tandon. Effect of sieved buckwheat (Fagopyrum esculentum) flour supplementation on lipid profile and glucose tolerance. Indian J Physiol Pharmacol 29 (1984) 69-74.
[3] R.L. Bijlani, B.M. Gandhi, M.C. Gupta, S. Manocha, and B.N. Tandon. Effect of whole buckwheat (Fagopyrum esculentum) flour supplementation on lipid profile & glucose tolerance. Indian J Med Res 81 (1985) 162-168.

[4] M. Dinu, D. Macchia, G. Pagliai, A.M. Gori, F. Cesari, et al. Symptomatic efficacy of buckwheat products in Non-Celiac Gluten Sensitivity (NCGS). Asia Pac J Clin Nutr 26 (2017) 630-636.
[5] G. Huang, M. Huang, W. Chen, Y. Huang, Z. Yang, and Y. You. Clinical effects of tartary buckwheat mixture on the treatment of early diabetic and nephropathy. J. Chin. Med. Mater 32 (2009) 1932-1935.
[6] A. Misan, A. Petelin, M. Stubelj, A. Mandic, O. Simurina, et al. Buckwheat - enriched instant porridge improves lipid profile and reduces inflammation in participants with mild to moderate hypercholesterolemia. Journal of Functional Foods 36 (2017) 186-194.
[7] M. Nishimura, T. Ohkawara, Y. Sato, H. Satoh, T. Suzuki, et al. Effectiveness of rutin-rich Tartary buckwheat (Fagopyrum tataricum Gaertn.) 'Manten-Kirari' in body weight reduction related to its antioxidant properties: A randomised, double-blind, placebo-controlled study. Journal of Functional Foods 26 (2016) 460-469.
[8] J. Qiu (a), Z. Li, Y. Qin, Y. Yue, and Y. Liu. Protective effect of tartary buckwheat on renal function in type 2 diabetics: A randomized controlled trial. Ther Clin Risk Manage 12 (2016) 1721-1727.
[9] J. Qiu (b), Y. Liu, Y. Yue, Y. Qin, and Z. Li. Dietary tartary buckwheat intake attenuates insulin resistance and improves lipid profiles in patients with type 2 diabetes: a randomized controlled trial. Nutr Res 36 (2016) 1392-1401.
[10] M. Shakib, S. Gabrial, and G. Gabrial. Buckwheat consumption improved lipid profile, fasting and postprandial blood glucose in hypercholesterolemic and type 2 diabetic patients. Int. J. Acad. Res. 3 (2011) 132-139.
[11] F. Sofi, L. Ghiselli, A. Whittaker, G. Pagliai, F. Cesari, et al. Consumption of buckwheat products and cardiovascular risk profile: A randomized single-blinded crossover trial. Nutr. Food Sci. 6 (2016) 1-8.
[12] E. Stokic, A. Mandic, M. Sakac, A. Misan, M. Pestoric, et al. Quality of buckwheat-enriched wheat bread and its antihyperlipidemic effect in statin treated patients. LWT 63 (2015) 556-561.
[13] D.M. Stringer, C.G. Taylor, P. Appah, H. Blewett, and P. Zahradka. Consumption of buckwheat modulates the post-prandial response of selected gastrointestinal satiety hormones in individuals with type 2 diabetes mellitus. Metab Clin Exp 62 (2013) 1021-1031.
[14] G. Wieslander, N. Fabjan, M. Vogrincic, I. Kreft, C. Janson, et al. Eating buckwheat cookies is associated with the reduction in serum levels of myeloperoxidase and cholesterol: a double blind crossover study in day-care centre staffs. Tohoku J Exp Med 225 (2011) 123-130.
[15] J. Zhao, and L. Guan. Effects of buckwheat flour eating for 8 weeks on blood glucose, serum lipids levels and blood pressure. Chin. J. Clin. Rehabil 7 (2003) 2231.
[16] G. Zheng, X. Pan, Z. An, and Y. Wang. Preliminary observation of lipid-decreasing effects of compound Tartary buckwheat flour on niddm patients. Beijing Med. J 5 (1991) 280-281.

Meta-analysis summary

Table S3. Meta-analysis of randomized clinical trials comparing buckwheat supplementation versus control

Outcome	Number. of unique studies	Size of the intervention arm	WMD (95% CI)	I^2	P value for heterogeneity	P-value for publication bias
**Blood lipids**						
Total cholesterol, *mmol/L*	8	220	-0.14 (-0.30; 0.02)	83.4%	0.002	0.67
Triglycerides, *mmol/L*	8	220	-0.02 (-0.15; 0.11)	85.5%	<0.001	0.20
HDL, *mmol/L*	8	220	-0.04 (-0.09; 0.02)	78.3%	0.03	0.78
LDL, *mmol/L*	7	200	-0.03 (-0.02; 0.16)	89.4%	<0.001	0.98
**Body morphology**						
Weight, *kg*	3	165	-0.14 (-1.50; 1.23)	0%	0.99	NA
**Glucose Homeostasis**						
Glucose, *mmol/L*	6	139	-0.18 (-0.36; 0.003)	87.7%	0.001	0.26

Note: WMD: Weighted mean difference I^2: variation across studies that is due to heterogeneity rather than chance; NA: Not applicable.

Table S4. Sensitivity analysis of the meta-analysis of randomized clinical trials comparing buckwheat supplementation versus control on blood lipids

Outcome	Number of unique studies	WMD (95% CI)	I^2	P value for heterogeneity	P-value for meta regression
**Total Cholesterol**					
Location					
Europe	4	**-0.28 (-0.37; -0.187)**	0%	0.57	0.59
Non-Europe	4	0.01 (-0.18; 0.21)	57.8%	0.07	
Trial duration					
≤5 weeks	3	-0.15 (-0.54; 0.24)	85.5%	0.001	0.57
>5 weeks	5	-0.14 (0.32; 0.03)	50.8%	0.09	
Females (%)					
≤50%	3	0.02 (-0.29; 0.32)	71.7%	0.03	0.56
>50%	5	**-0.23 (-0.41; -0.05)**	59.6%	0.04	
Health status					
Healthy	4	-0.08 (-0.23; 0.07)	12.6%	0.33	0.78
Unhealthy	3	**-0.28 (-0.37; -0.19)**	69.1%	0.002	
Mix*	1	0.23 (-0.02; 0.48)	NA	NA	
Publication year					
≤2000	**2**	-0.11 (-0.46; 0.24)	19.5%	**0.27**	0.38
>2000	6	-0.15 (-0.34; 0.04)	76.4%	0.001	
**Triglycerides**					
Location					
Europe	4	-0.05 (-0.22; 0.11)	85.3%	0.09	0.83
Non-Europe	4	0.015 (-0.22; 0.24)	53.1%	<0.001	
Trial duration					
≤5 weeks	3	0.05 (-0.16; 0.26)	61.4%	0.08	0.60
>5 weeks	5	-0.07 (-0.24; 0.11)	80.9%	<0.001	

Outcome	Number of unique studies	WMD (95% CI)	I²	P value for heterogeneity	P-value for meta regression
Females (%)					
≤50%	3	-0.02 (-0.36; 0.32)	88.6%	<0.001	0.68
>50%	5	0.02 (-0.10; 0.13)	47.3%	0.11	
Health status					
Healthy	3	-0.11 (-0.43; 0.21)	87.3%	<0.001	0.98
Unhealthy	4	-0.01 (-0.15; 0.13)	43.5%	0.15	
Mix	1	**0.19 (0.03; 0.35)**	NA	NA	
Publication year					
≤2000	2	**-0.28 (-0.43; -0.13)**	0%	0.53	0.66
>2000	6	0.08 (-0.001; 0.16)	29.6%	0.21	
**HDL Cholesterol**					
Location					
Europe	4	-0.04 (-0.09; 0.02)	72.4%	0.01	0.90
Non-Europe	4	-0.02 (-0.06; 0.02)	0%	0.93	
Trial duration					
≤5 weeks	3	-0.002 (-0.05; 0.02)	13.9%	0.31	0.77
>5 weeks	5	**-0.09 (-0.14; -0.04)**	6.4%	0.37	
Females (%)					
≤50%	3	-0.01 (-0.06; 0.03)	0%	0.91	0.56
>50%	5	-0.05 (-0.15; 0.04)	65.6%	0.02	
Health status					
Healthy	3	-0.03 (-0.11; 0.04)	0%	0.88	0.78
Unhealthy	4	-0.04 (-0.14; 0.06)	80.6%	0.001	
Mix	1	-0.03 (-0.14; 0.05)	NA	NA	
Publication year					

Outcome	Number of unique studies	WMD (95% CI)	I^2	P value for heterogeneity	P-value for meta regression
≤2000	**2**	-0.03 (-0.17; 0.11)	0%	0.45	0.41
>2000	6	-0.04 (-0.09; 0.02)	67.5%	0.009	

* 'Mix' category indicates those trials that included both healthy and unhealthy subjects.

Note: WMD: Weighted mean difference I^2: variation across studies that is due to heterogeneity rather than chance

Statistically significant values are bolded.

Methodological quality assessment

Figure S1. Methodological quality of included nonrandomized studies based on ROBINS-I tool

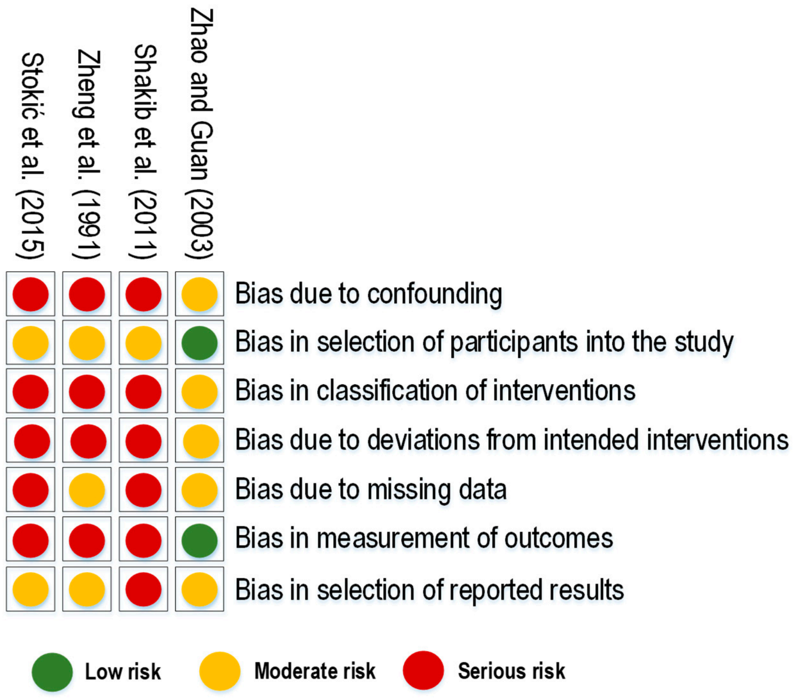

Figure S2. Methodological quality of included randomized controlled trials based on Cochrane's Collaboration Tool (RoB2) for randomized controlled trials

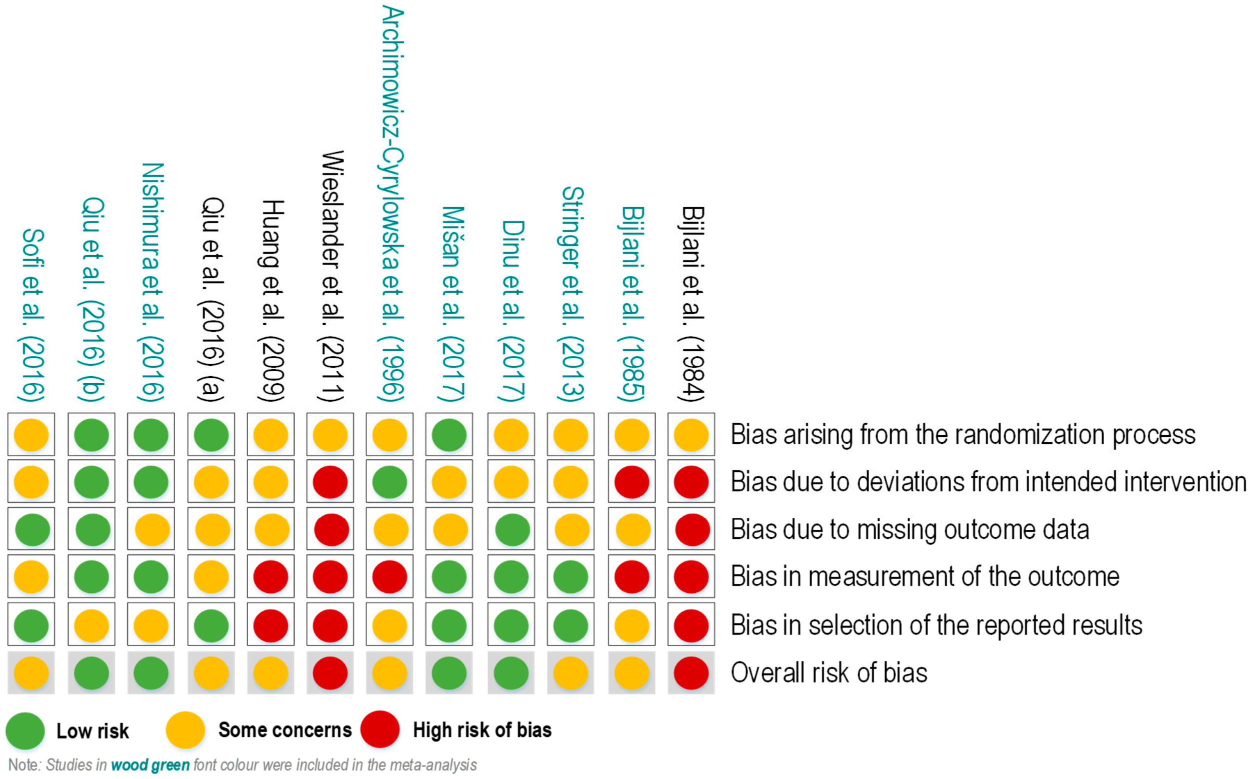

Publication bias

Figure S3. Publication bias of meta-analysis of buckwheat and total cholesterol

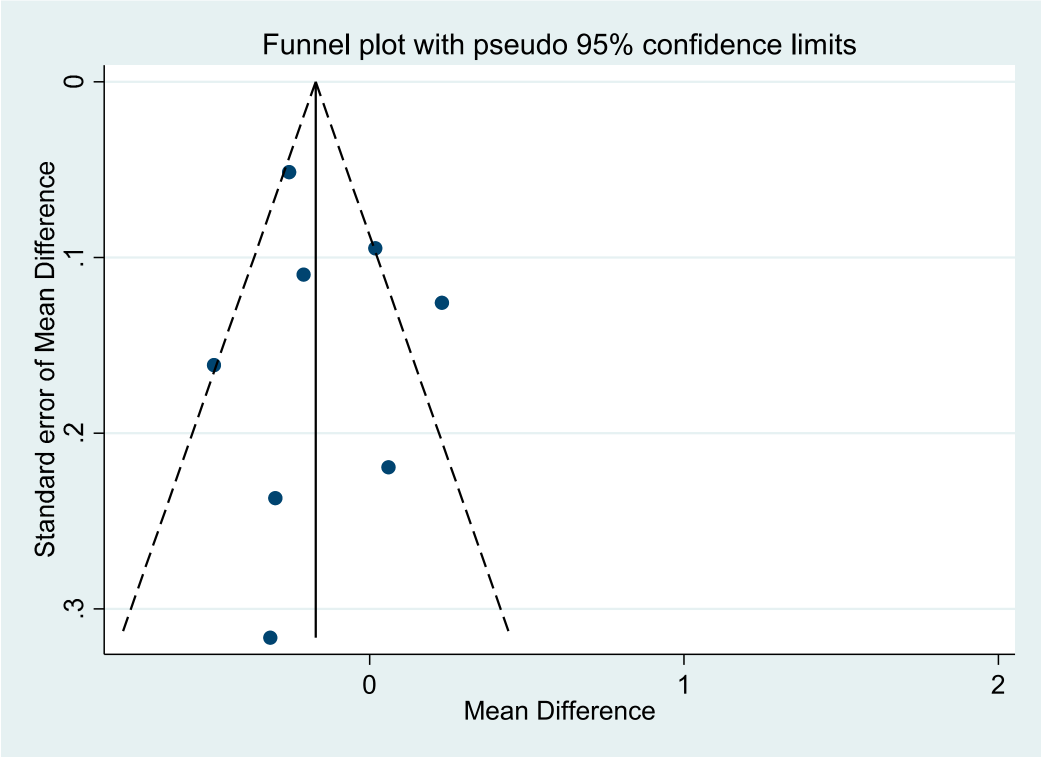

Figure S4. Publication bias of meta-analysis of buckwheat and triglycerides

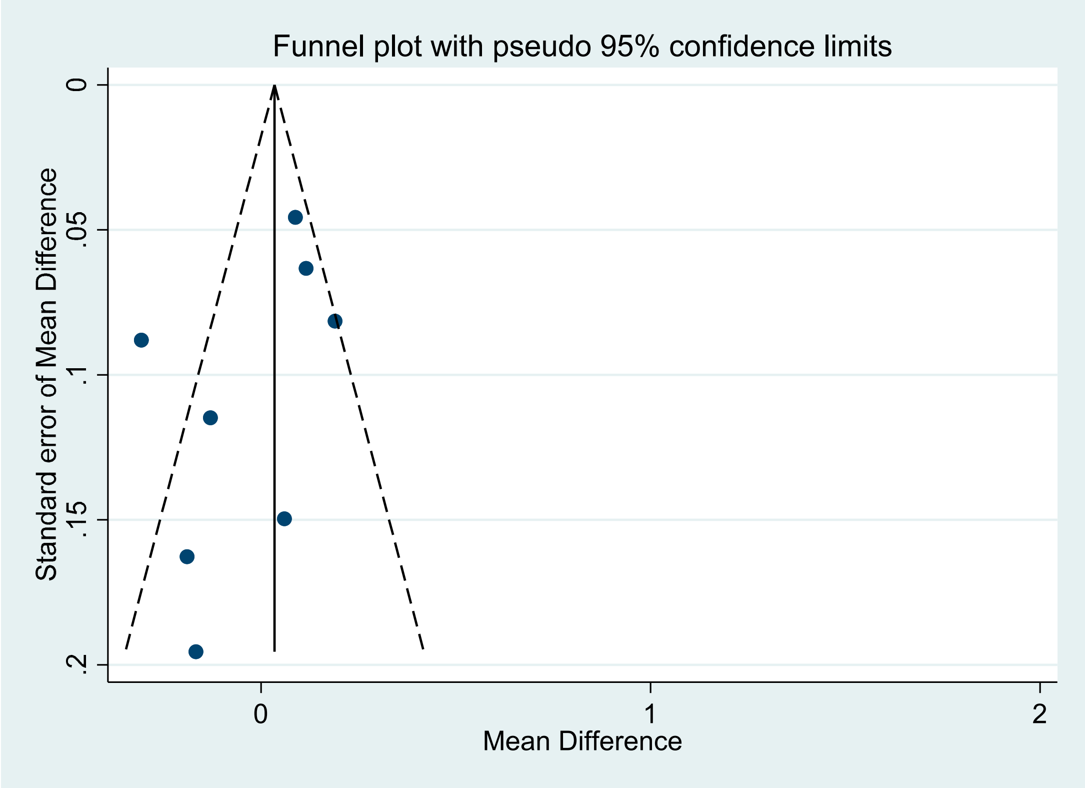

Figure S5. Publication bias of meta-analysis of buckwheat and HDL

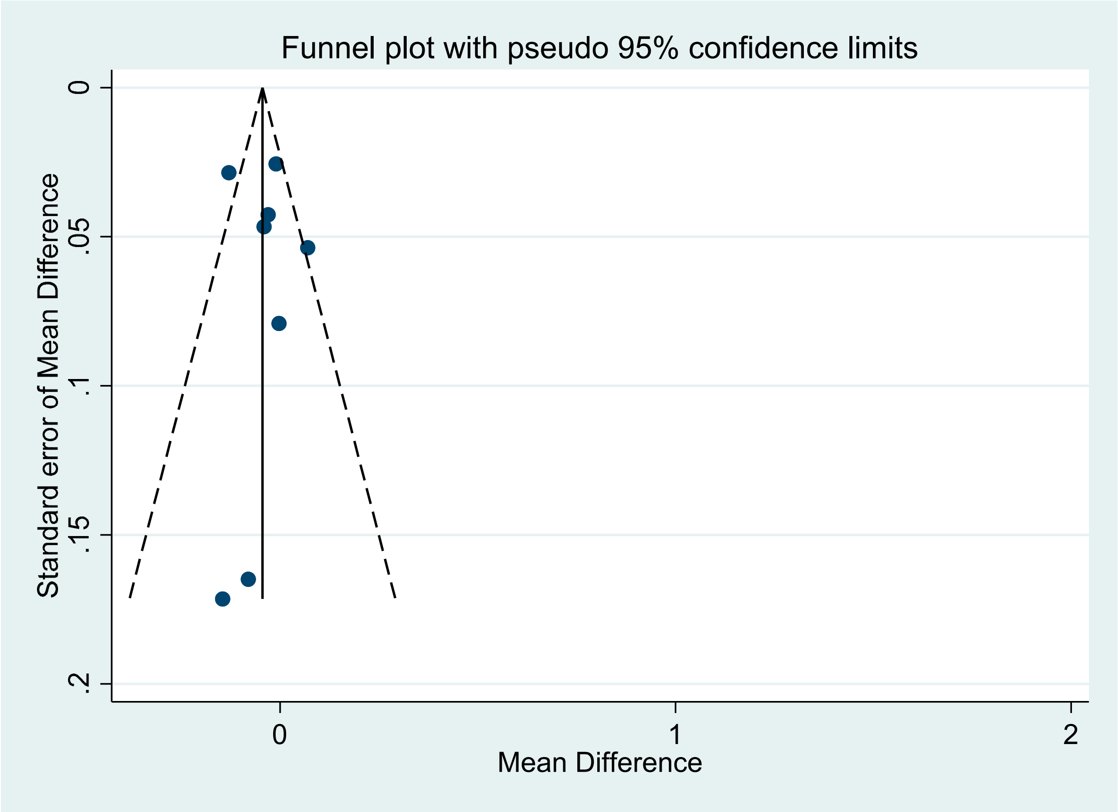

Figure S6. Publication bias of meta-analysis of buckwheat and LDL

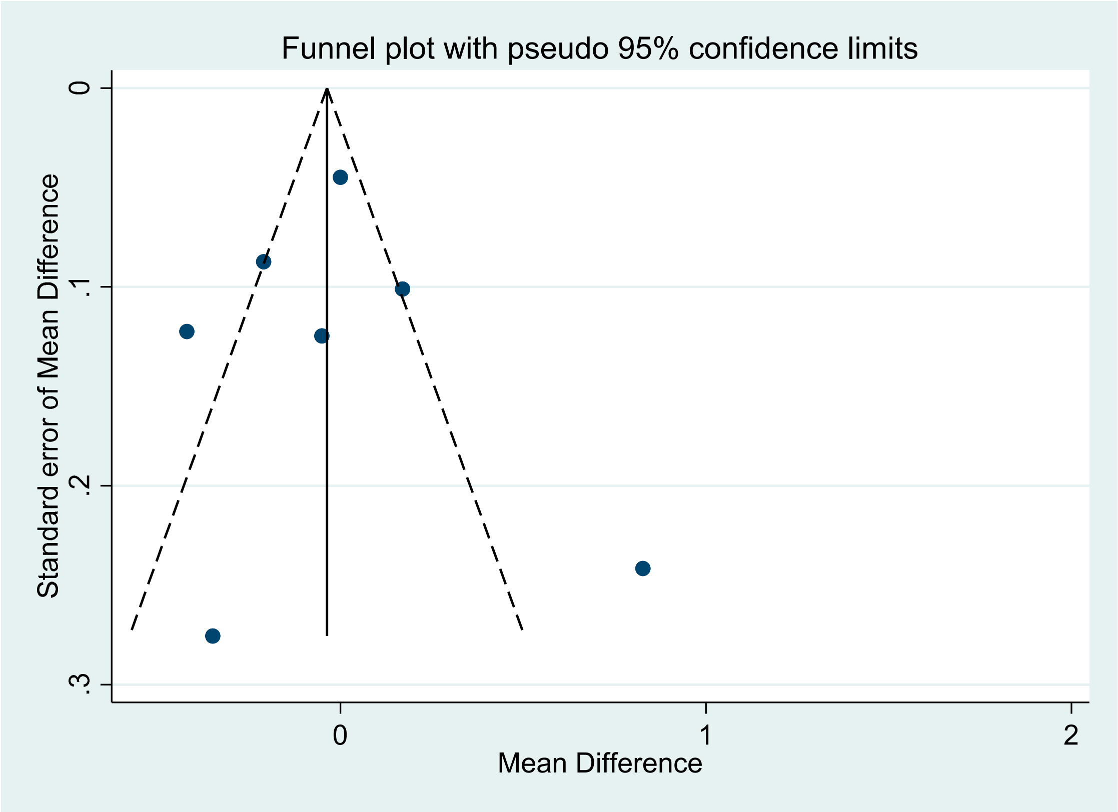

Figure S7. Publication bias of meta-analysis of buckwheat and glucose

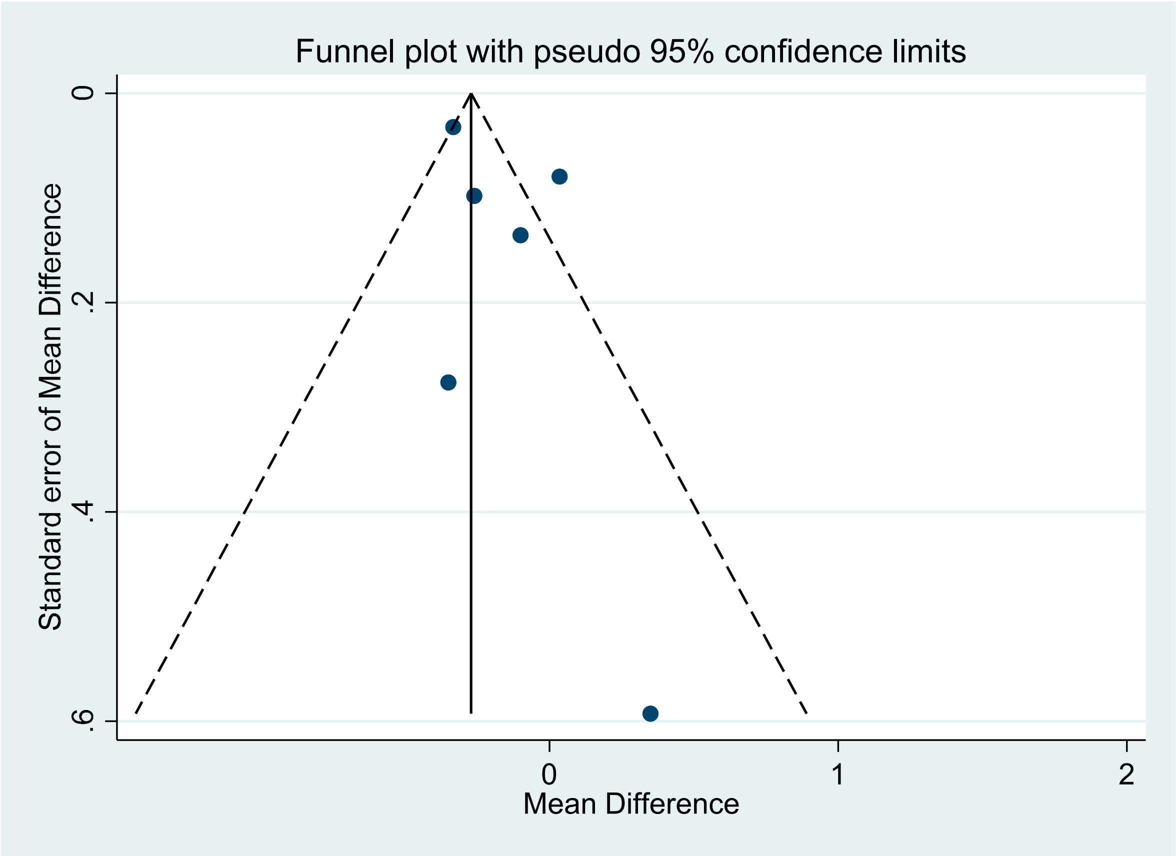
Funnel plot with pseudo 95% confidence limits
Standard error of Mean Difference
0
.2
.4
.6
0
1
2
Mean Difference

Sensitivity analysis of the meta-analysis on total cholesterol after removing the non-grain component study

Figure S8. Sensitivity analysis of the meta-analysis on total cholesterol after removing the non-grain component study (i.e. Archimowicz-Cyrylowska et al. (1996))

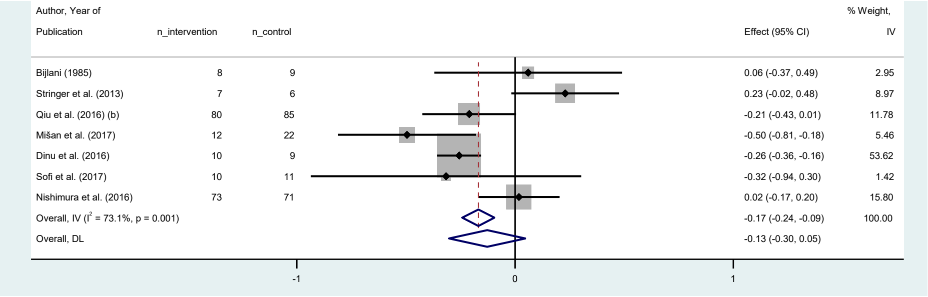

Figure S9. Sensitivity analysis of the meta-analysis on triglycerides after removing the non-grain component study (i.e. Archimowicz-Cyrylowska et al. (1996))

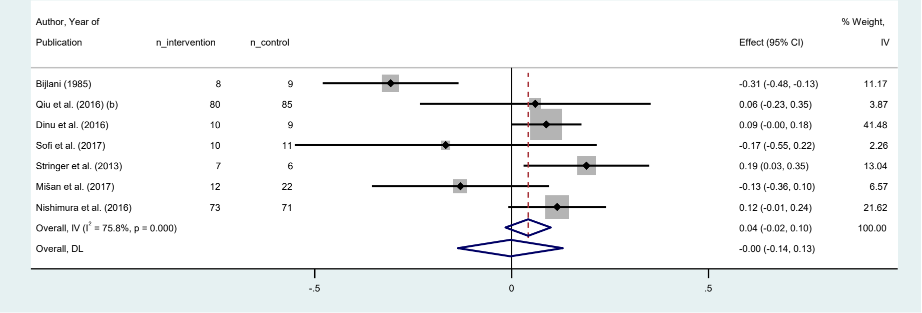

Figure S10. Sensitivity analysis of the meta-analysis on HDL cholesterol after removing the non-grain component study (i.e. Archimowicz-Cyrylowska et al. (1996))

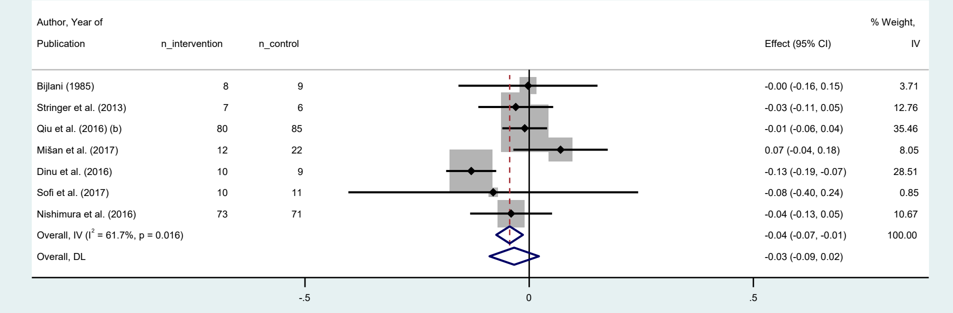

Figure S11. Sensitivity analysis of the meta-analysis on glucose after removing the non-grain component study (i.e. Archimowicz-Cyrylowska et al. (1996))

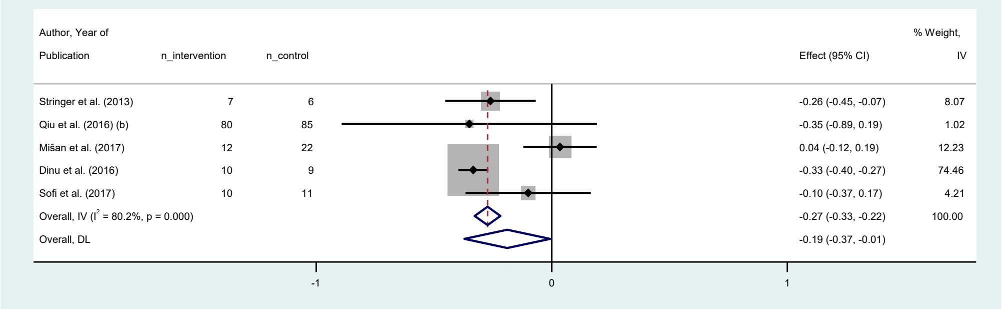

Table S5. Lipid profile markers at baseline

Author, Year of Publication	Outcome	Unit	Intervention arm size	Mean value at baseline
Bijlani (1985)	LDL cholesterol	mmol/L	8	2.08
Stringer et al. (2013)	LDL cholesterol	mmol/L	7	2.65
Qiu et al. (2016) (b)	LDL cholesterol	mmol/L	80	3.00
Mišan et al. (2017)	LDL cholesterol	mmol/L	12	4.55
Dinu et al. (2016)	LDL cholesterol	mmol/L	10	2.89
Sofi et al. (2017)	LDL cholesterol	mmol/L	10	3.14
Nishimura et al. (2016)	LDL cholesterol	mmol/L	73	4.07
Bijlani (1985)	HDL cholesterol	mmol/L	8	1.02
Stringer et al. (2013)	HDL cholesterol	mmol/L	7	1.25
Qiu et al. (2016) (b)	HDL cholesterol	mmol/L	80	1.20
Mišan et al. (2017)	HDL cholesterol	mmol/L	12	1.37
Dinu et al. (2016)	HDL cholesterol	mmol/L	10	1.86
Sofi et al. (2017)	HDL cholesterol	mmol/L	10	1.62
Nishimura et al. (2016)	HDL cholesterol	mmol/L	73	1.87
Archimowicz-Cyrylowska et al. (1996)	HDL cholesterol	mmol/L	20	1.16
Bijlani (1985)	Triglycerides	mmol/L	8	1.00
Qiu et al. (2016) (b)	Triglycerides	mmol/L	80	2.10
Dinu et al. (2016)	Triglycerides	mmol/L	10	0.95
Sofi et al. (2017)	Triglycerides	mmol/L	10	1.28
Archimowicz-Cyrylowska et al. (1996)	Triglycerides	mmol/L	20	1.64
Stringer et al. (2013)	Triglycerides	mmol/L	7	1.27
Mišan et al. (2017)	Triglycerides	mmol/L	12	1.89
Nishimura et al. (2016)	Triglycerides	mmol/L	73	0.95
Bijlani (1985)	Total cholesterol	mmol/L	8	4.55
Stringer et al. (2013)	Total cholesterol	mmol/L	7	4.27
Qiu et al. (2016) (b)	Total cholesterol	mmol/L	80	5.08
Mišan et al. (2017)	Total cholesterol	mmol/L	12	6.82
Dinu et al. (2016)	Total cholesterol	mmol/L	10	5.33
Sofi et al. (2017)	Total cholesterol	mmol/L	10	5.23
Nishimura et al. (2016)	Total cholesterol	mmol/L	73	6.25
Archimowicz-Cyrylowska et al. (1996)	Total cholesterol	mmol/L	20	5.70

Note: Mean values at baseline that were out of normal range are highlighted in grey
Ranges considered normal: LDL cholesterol: <3.4 mmol/L; Total cholesterol: <5.2 mmol/L; HDL cholesterol: >1.6 mmol/L; Triglycerides: <1.7 mmol/L.

Leave-one-out analysis

Figure S12. Leave one out sensitivity analysis for total cholesterol

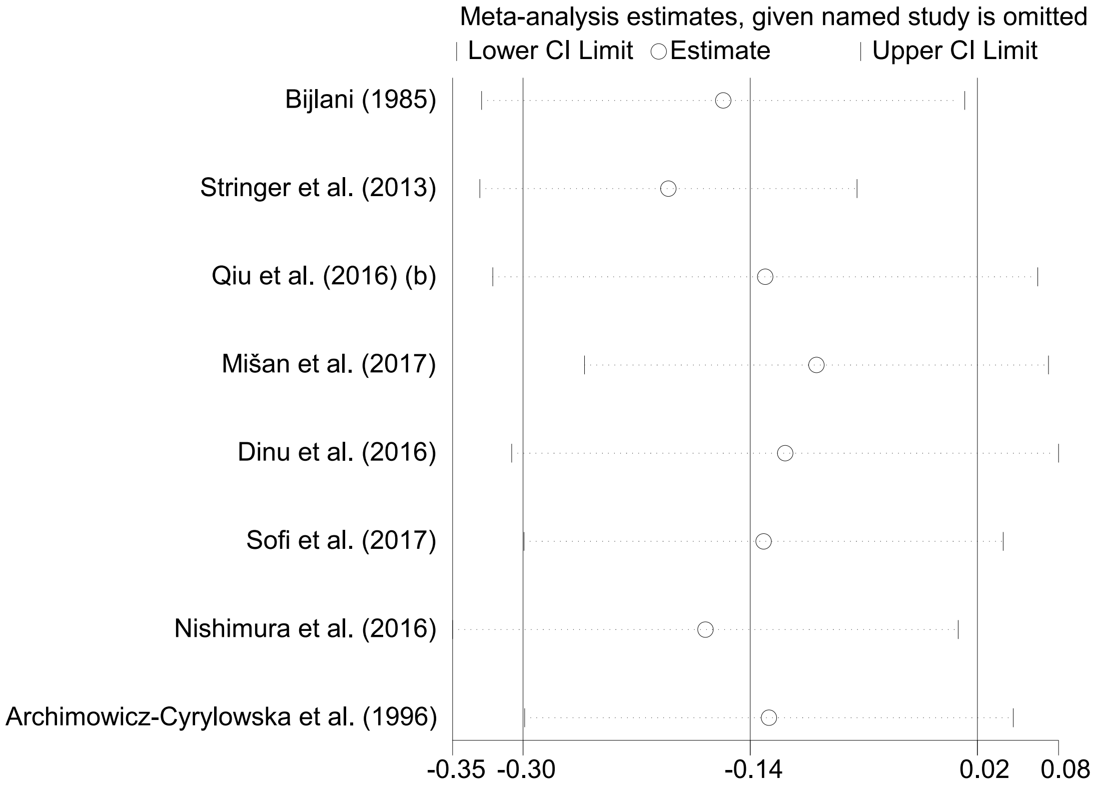

Note: Removing stringer et al makes it significant beneficial -0.20 (-0.33; -0.065)

Figure S13. Leave one out sensitivity analysis for triglycerides

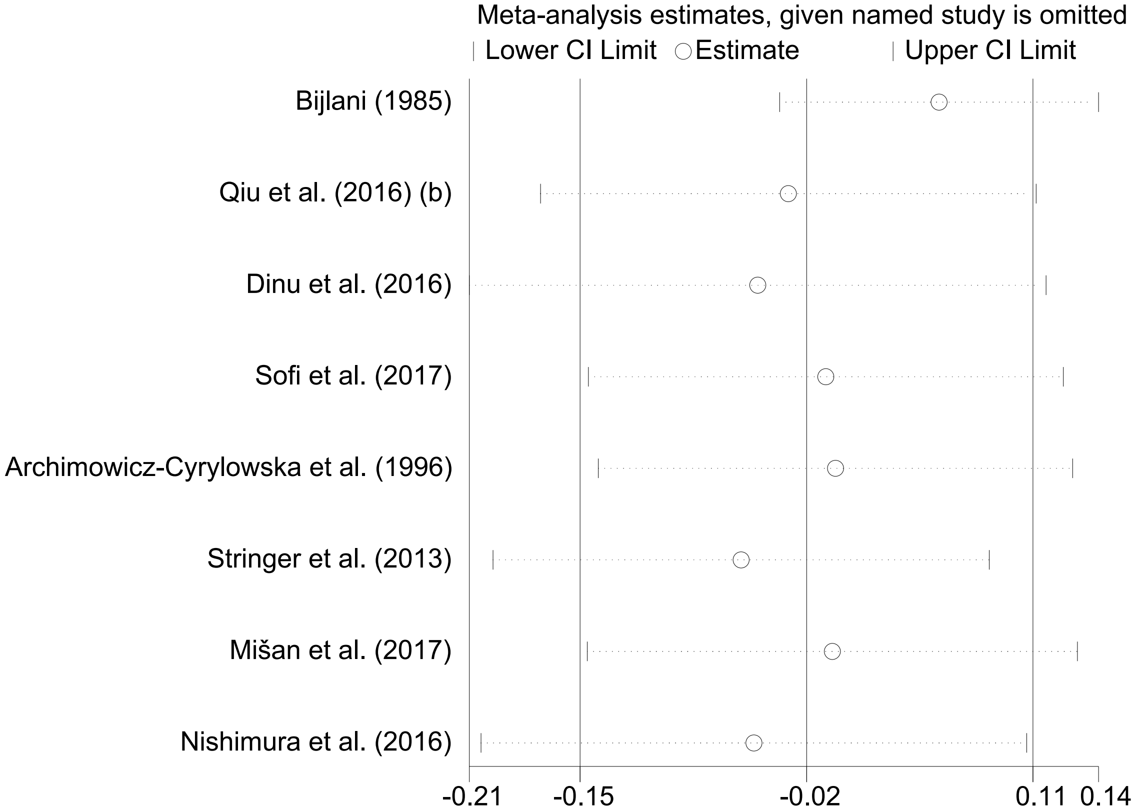

Note: There was no difference in the overall effect for any study

Figure S14. Leave one out sensitivity analysis for HDL Cholesterol

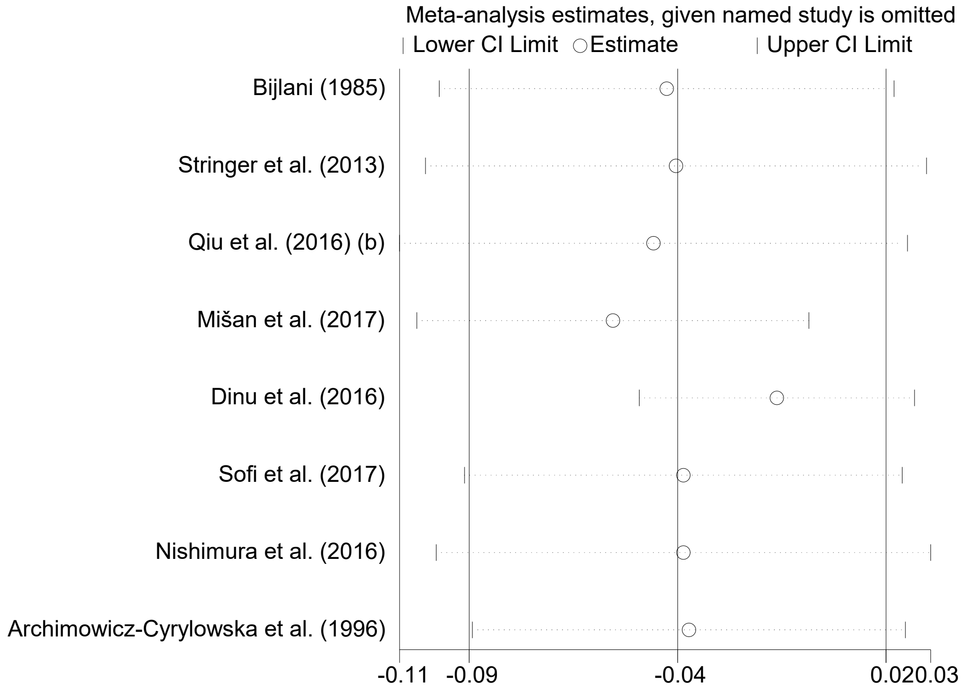

Note: Removing Mišan et al generated a significant beneficial effect on HDL cholesterol: -0.05 (-0.10; -0.03)

Figure S15. Leave one out sensitivity analysis for LDL Cholesterol

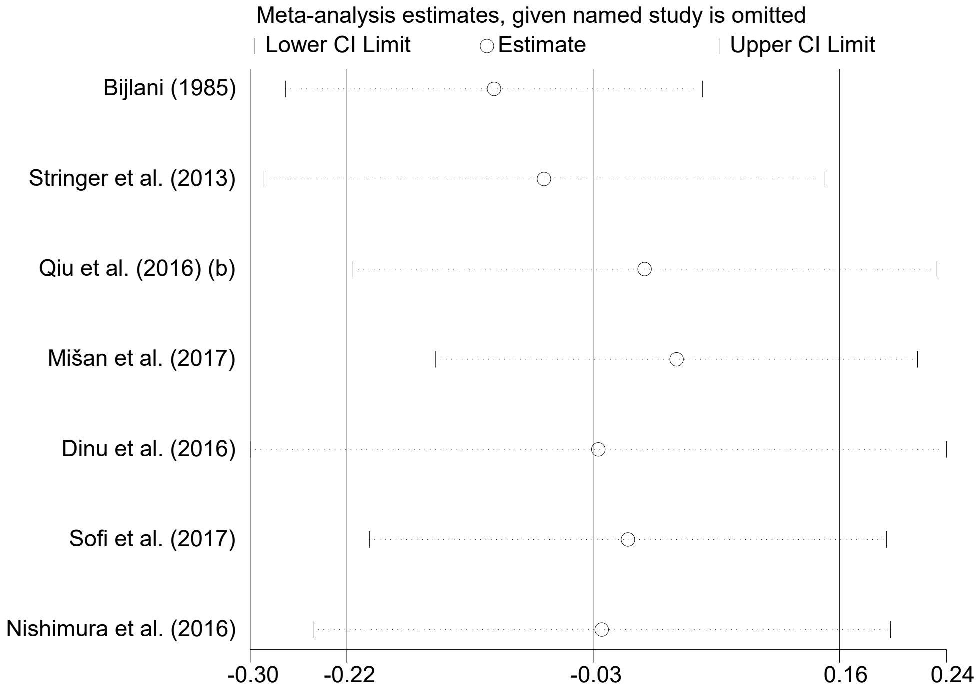

Note: There was no difference in the overall effect for any study.

Figure S16. Leave one out sensitivity analysis for glucose

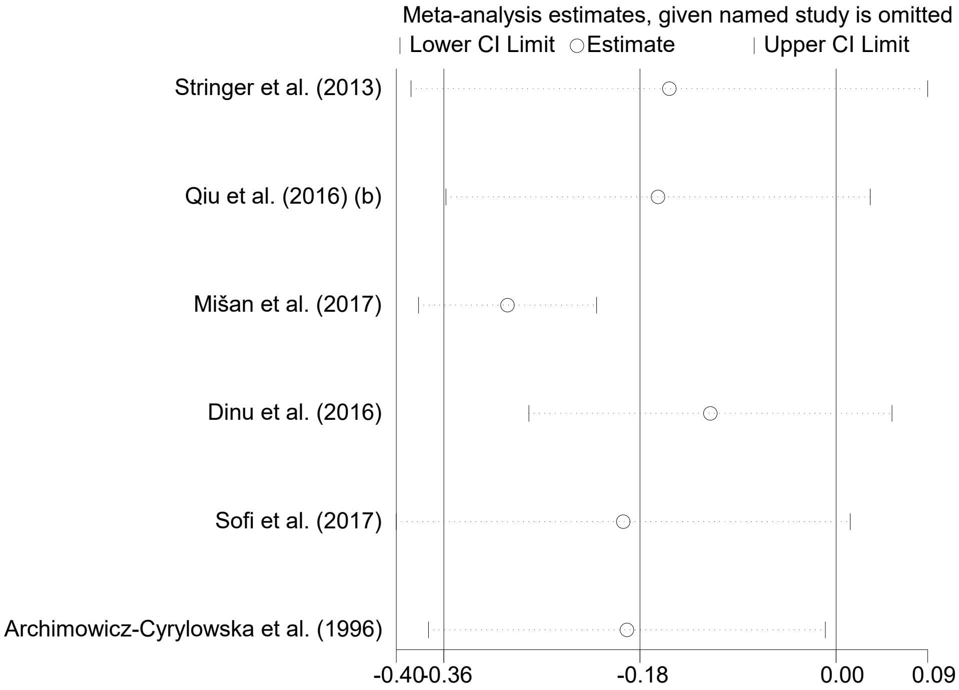

Note: Removing Mišan et al significant beneficial: -0.30; -0.38; -0.22
